# Unable or Unwilling to Exercise Self-control? The Impact of Neuroscience on Perceptions of Impulsive Offenders

**DOI:** 10.3389/fpsyg.2017.02189

**Published:** 2018-01-04

**Authors:** Robert Blakey, Tobias P. Kremsmayer

**Affiliations:** ^1^Centre for Criminology, University of Oxford, Oxford, United Kingdom; ^2^Department of Communication Sciences & Disorders, Bouvé College of Health Sciences, Northeastern University, Boston, MA, United States

**Keywords:** neurocriminology, public perceptions, moral attitudes, moral responsibility, blame attribution

## Abstract

In growing numbers of court cases, neuroscience is presented to document the mental state of the offender at the level of the brain. While a small body of research has documented the effects of describing the brain state of psychotic offenders, this study tested the impact of neuroscience that could apply to far more offenders; that is the neuroscience of impulse control. In this online vignette experiment, 759 participants sentenced a normally controlled or normally impulsive actor, who committed a violent offense on impulse, explained in either cognitive or neurobiological terms. Although participants considered the neurobiological actor less responsible for his impulsive disposition than the cognitive actor, the neuroscientific testimony did not affect attributions of choice, blame, dangerousness, or punishment for the criminal act. In fact, the neuroscientific testimony exacerbated the perception that the offender offended consciously and “really wanted” to offend. The described disposition of the actor was also influential: participants attributed more capacity for reform, more free choice and consequently, more blame to the normally controlled actor. Participants also attributed this actor's offending more to his social life experiences and less to his genes and brain. However, this shift in attributions was unable to explain the greater blame directed at this offender. Together, such findings suggest that even when neuroscience changes attributions for impulsive character, attributions for impulsive offending may remain unchanged. Hence this study casts doubt on the mitigating and aggravating potential of neuroscientific testimony in court.

## Introduction

The retributive justification for punishment requires that sentences are tailored to the seriousness of the offense and the culpability of the offender (von Hirsch, 1976). The law does not stipulate a formula for blame; instead, judges assess culpability from a range of legally eligible mitigating and aggravating factors. In determining the eligibility and weight attributed to such factors, judges are guided by the legal definition of responsibility (Morse, 2006). While the definition of legal responsibility is therefore critical to the implementation of retributive punishment, the definition is not entirely clear. It is traditionally claimed that legal attributions of responsibility reflect the general capacity of the offender to exercise rational choice. The capacity to exercise rational choice comprises the capacity to distinguish reality from fantasy, right from wrong, to form intent that is consistent with desire and to act upon that intent by exercising self-control over impulses (Morse, 2004a,b, 2008).

While the capacity for rational choice is the only stipulated criterion for legal responsibility, law makers may have intended a rational choice to mean a free choice that is not deterministically caused, yet simply never conceived that intentional choices could be deterministically caused (Kolber, 2016). This argument is consistent with the claim that people exclusively attribute moral responsibility to free, not simply rational, offenders:

“The law's exclusive interest in rationality misses something intuitively important. In our opinion, rationality is just a presumed correlate of what most people really care about…[that being the ultimate cause of criminal behaviour]” (Greene and Cohen, 2004, p. 1780).

The current study aimed to test whether this claim accurately characterizes lay intuitions about self-control, as a component of rational choice. We predicted that lay people do care about the ultimate cause of self-control, yet only after first learning that the offender has an impulsive *disposition*. In this context, people do care about the cause of irrationality, yet that *is* a legally relevant concern. Contrary to Greene and Cohen, this study predicts there to be no discrepancy between intuitive and legal judgements of culpability.

### Is the impulsive actor unable or unwilling to exercise self-control?

Some people's impulsive actions reflect their impulsive characters. When judging this type of person (hereon “the impulsive actor”), one could attribute their impulsive disposition to one of two factors: an incapacity to exert self-control for uncontrollable reasons or an unwillingness to exert self-control for controllable reasons (Steinberg, 2009). Given its persistence, the impulsivity either appears to be involuntary and so less deserving of punishment (von Hirsch, 2009), or to be the product of greater intent and so more deserving of punishment, since the offender continues to offend despite gaining knowledge of the causes and consequences of the behavior (Roberts, 2009). Hence it is possible to adopt either a mitigating or an aggravating interpretation of the stable disposition to commit impulsive acts.

### The neuroscientific response

Neuroscience may play a pivotal role in determining which interpretation of stable impulsivity people adopt. The exercise of self-control is also a brain mechanism and brain function is ultimately the product of uncontrollable influences (Steinberg, 2013). Therefore, the degree of self-control used in a particular moment reflects the neurobiological capacity for self-control in that particular moment. Hence the impulsive offender is simply a product of his capacities and for this reason, does *not* deserve punishment.

### The legal response

It is legally important to distinguish an unwillingness to exercise rational choice in a particular moment from a general incapacity to exercise rational choice (Goldstein et al., 2003); only the latter justifies a reduction in the legal responsibility (Steinberg, 2009). For example, whereas a psychotic offender *could not* have avoided hallucinating, an intoxicated offender could have avoided alcohol consumption. Therefore, the psychotic offender lacks the capacity to exercise rational choice, whereas the intoxicated offenders only lacks the willingness. Hence there is a far stronger legal argument for excusing the psychotic offender. Accordingly, the neuroscience of impulsivity could derive legal relevance from the assumption that it is documenting the capacity, rather than the motivation, to exercise rational choice.

### The intuitive response in theory

When asked to sentence a hypothetical offender, people tailor their sentence recommendations to the capacity of the offender to exercise rational choice, considering both his intentionality (e.g., Darley et al., 2000) and whether that intent was coerced or reflects a genuine desire to offend (e.g., Woolfolk et al., 2006). The online study tested whether lay judgements also vary with a third, more contested, component of the capacity to exercise rational choice: the capacity to exercise self-control. In this study, the participants received no *direct* information about the *capacity* of the offender to exercise self-control, since such information is also unavailable in real life: self-report, observational, cognitive, and neuroscientific measures only provide correlational, not causal, indicators of self-control. Hence, to some extent, it is always a matter of interpretation whether one considers those indicators to reveal the capacities or the motivations of the offender.

In the online study, participants read about two indicators of self-control. First, participants learnt whether the offender *normally* exercised self-control. People attribute dispositional causes to behaviors that persist across time and situations (Kelley, 1973). Hence the stability of the behavior might be considered one cue that the offender lacked the capacity, rather than the motivation, to exercise self-control. Second, the vignette attributed the disposition of the offender either to himself or to his brain. Neuroscience is premised on physicalism; the idea that everything is represented in physical matter, including all aspects of the mind. From this perspective, there is no difference between the offender and his brain: “all psychological states are also biological ones” (Monterosso and Schwartz, 2012). In contrast, lay people tend to intuitively adopt the conception that brain and mind are at least partially separate (Bloom, 2004; Forstmann and Burgmer, 2015). Therefore, people may distinguish the brain and the person as sources of self-control and infer that the brain more faithfully represents the *capacity* of the offender to exercise self-control.

There is currently only indirect evidence that people perceive the brain to be a physical manifestation of cognitive capacities rather than motivations. People are less likely to attribute moral responsibility to offenders when the brain is presented as an explanation of every behavior (Nahmias et al., 2007) or a specific mental disorder (e.g., Aspinwall et al., 2012). Hence these offenders are presumably less likely to be considered *capable* of rational choice. The traits that people believe to be more attributable to the brain are also the traits that people believe to be better indicators of moral *character* (Fernandez-Duque and Schwartz, 2016). Therefore, people conceive the brain to be the source of stable behaviors. In turn, this conception could result from, or lead to, the perception that the brain houses capacities rather than motivations. Consequently, in the courtroom, neuroscience might be used to strengthen the argument that the offender is *unable*, rather than simply unwilling, to exercise rational choice; the online study tested whether the science would do so successfully.

### The intuitive response in practice

Some mock court studies have found that people are less likely to attribute moral responsibility to the offender whose mental illness is described in neurobiological, rather than cognitive, terms (Gurley and Marcus, 2008; Schweitzer and Saks, 2011; Schweitzer et al., 2011; Aspinwall et al., 2012; Greene and Cahill, 2012) and in real court cases (Denno, 2015). More generally, people find cognitive explanations more compelling when those explanations are supplemented with irrelevant neuroscience (Weisberg et al., 2008, 2015), even after controlling for the jargon and the status of neuroscience (Fernandez-Duque et al., 2015). Such evidence supports the potential for neuroscience to exert a mitigating influence in court.

In the previously mentioned mock court studies, neuroscience was used to explain severe mental illness rather than an impulsive character. Yet two studies have addressed the impact of describing impulsivity in neurobiological terms, with further studies presenting *genetic* testimony of the trait. The two studies found no effect of neuroscience on sentencing in non-capital cases (Appelbaum et al., 2015; Scurich and Appelbaum, 2016). However, neuroscientific testimony did reduce the probability of participants deciding upon the death penalty in a capital case—an effect that was not found with neuro*genetic* testimony (Appelbaum et al., 2015). Although the authors did not explicitly refer to impulsivity, Monterosso et al. (2005) presented a neurochemical explanation of a violent murder that was disproportionate to the source of provocation. In comparison to child abuse, the neurochemical explanation reduced attributions of blame, voluntariness, and punitiveness, also increasing sympathy for the offender; the offending was reconceived as “automatic and uncontrollable” (p. 143). Hence neurobiological accounts of impulsivity may have mitigating effect.

Evidence for the influence of genetic testimony is more limited. Research has found no effect of genetic testimony on sentencing decisions and insanity verdicts in non-capital cases and capital cases (Appelbaum et al., 2015), including attributions of responsibility and punishment for second-degree murder (Appelbaum and Scurich, 2014) and for non-criminal deviance, where one might expect less emotional resistance to mitigation (Scurich and Appelbaum, 2016). Cheung and Heine (2015) compared genetic and environmental explanations of the dispositional tendency to respond violently to provocation. The genetic testimony reduced the perceived extent to which the offender “had conscious control over his actions” and “intended to kill the victim” (p. 1725), while increasing the perceived applicability of the Insanity and Diminished Capacity defenses. However, categorical decisions on the defenses, attributions of “criminal responsibility” and sentences were unaffected by the genetic testimony (studies one and two). Although reduced attributions of “conscious control” predicted shorter prison sentences, the increased attribution of internal causes and reoffending predicted longer sentences; hence there was no net effect of genetic testimony on sentence length. In study three, however, attributions of criminal responsibility and recommended sentences were reduced, with the use of a more sensitive sentencing measure.

The findings of Cheung and Heine (2015) indicate that the mitigating potential of biological criminology can be squashed by simultaneous increases in the attribution of dangerousness; in other words, by consequentialist responses to the science. Hence one cannot infer that neuroscience lacks any mitigating potential from evidence that the ultimate sentence is unaffected by neuroscientific testimony (Appelbaum et al., 2015; Scurich and Appelbaum, 2016). Therefore, the online study measured various retributive concerns regarding the offender in the vignette.

### Is a lapse from high self-control freely willed or deterministically caused?

Thus, far I have discussed the impulsive actor. However, not only impulsive people commit impulsive acts; normally controlled people can commit impulsive offenses too. The fact that such people normally exercise self-control clearly indicates their general capacity to exercise self-control. Hence one might consider this type of offender able, yet unwilling, to exercise self-control on this particular occasion. In turn, this offender might be attributed complete moral responsibility, regardless of their reason for deviating from self-control on this occasion.

### The neuroscientific response

Neuroscientists would attribute a lapse in controlled behavior to a lapse in normal brain function; in turn, this deviation in brain activity would be attributed to random chance variation or to an unusually tempting feature of the situation faced by the actor (Greene and Cohen, 2004). The brain can still account for deviations in behavior, since brain activity also deviates by chance and with changes to sensory input from the external situation; which results from a chain of previous environmental events. Therefore, from a neuroscientific perspective, the controlled actor who lapses into impulsive offending has no ultimate control over the process of random chance or the chain of environmental events that creates the unusually tempting situation. If the lapse in controlled behavior is caused in this manner, the controlled actor does not *deserve* punishment for the lapse.

### The legal response

One day people may accept that every moral failure is a moral weakness: people fail to exercise self-control because of their brain, and in turn, because of genetic and environmental factors that affect the brain. Even if such a day manifested, it is typically argued that offenders would remain legally responsible for their unfree actions (Morse, 2004a). Offenders are *legally* responsible for their actions so long as their mind is generally capable of rational choice, even if every choice is ultimately caused by uncontrollable genetic and environmental factors, and in this sense, is unfree (Morse, 2006). Since evidence of causation is therefore compatible with legal attributions of responsibility, the law can be described as compatibilist. Hence neurocriminology does not invalidate the legal justification for retributive punishment.

No statute or case law *explicitly* endorses this legal attribution of responsibility to the unfree, yet rational, offender (Kolber, 2016). On paper, to be held responsible, the law only requires offenders to have sufficient self-control to be capable of acting upon desired intentions (Morse, 2008). However, centuries ago, the law-makers may have simply assumed that the degree of self-control a person exercises is a free choice that is not deterministically caused and therefore, a decision that is never reducible to a flash of brain activity, caused by a chain of events going back to birth (Kolber, 2016). In other words, people may have traditionally considered self-control to always be a motivation, never a capacity. The founders of retributive punishment, therefore, “plausibly never intended to punish people who make decisions in the mechanistic manner scientists now take to characterize human choice” (Kolber, 2016, p. 3). If evidence of causation was incompatible with legal attributions of responsibility, the law would be described as incompatibilist. In this novel interpretation, neurocriminology does invalidate the legal justification for retributive punishment.

### The intuitive response

When the impulsive offender was described as a normally controlled actor (hereon “the controlled actor”), participants read that either the actor or his brain was responsible for his normally controlled behavior. When participants read that his brain was responsible, they might also attribute the criminal *lapse* in self-control to the brain. Yet still, this may pose no implications for their attributions of blame and punishment, given the philosophical evidence that lay people consider causation and blame to be compatible (e.g., Nahmias et al., 2005). Hence, even if the lapse is conceived as caused (rather than willed), the intuitive drive for retributive punishment may remain intact. In this respect, the legal and intuitive responses may be remarkably similar.

In sum, lay attributions of moral responsibility may persist for the controlled actor who *lapses into* impulsivity: his previously controlled behavior indicates his general capacity to exercise self-control, rendering him (legally and intuitively) responsible for his lapse in self-control. I therefore expected participants to blame the controlled actor for offending, regardless of the neuroscientific testimony. In contrast, when the offender is a persistently impulsive actor, participants may be primed to consider the possibility that he lacks the *capacity* to exercise self-control. In this context, people may be sensitized to further capacity cues, such as neuroscientific evidence of impulsivity. Consequently, we expected participants to attribute less responsibility to the neurobiologically impulsive actor.

### Consequentialist justifications for punishment

While the focus of this study is the retributive justification for punishment, participants also answered questions about consequentialist concerns; those are the practical consequences of punishment, such as rehabilitation and deterrence (Carlsmith and Darley, 2008). Greene and Cohen (2004, p. 1784) predict that as a result of neuroscience, “retributivist notions of criminal responsibility…will give way to consequentialist ones,” causing offenders to be treated more “humanely.” Yet, given historical abuse of biological explanations (Rafter, 2008), other theorists fear that neuroscience will aggravate sentences by creating the impression that the offender is unchangeably dangerous (Snead, 2007; Dar-Nimrod and Heine, 2011). There is no evidence of this aggravating effect on judgements of psychopathic or schizophrenic offenders (Aspinwall et al., 2012; Greene and Cahill, 2012; Saks et al., 2014). Since, in the current study, the offender was merely described as impulsive, we also did not expect neuroscientific testimony to impact consequentialist concerns.

### Hypotheses

Participants read about a hypothetical offender, described as committing a violent offense on impulse. In the subsequent defense testimony for the case, participants read that the offender was normally impulsive or normally controlled, either in cognitive or neurobiological terms. First, it was hypothesized that the normal character of the offender would be considered relevant to retributive judgements:

H1: Participants would attribute more “free choice,” fault and punishment to the offender who was normally controlled (rather than normally impulsive).
  Second, it was hypothesized that the effect of neuroscientific testimony would be restricted to judgements of the normally impulsive actor:
H2: Participants would be more likely to believe the impulsive offender lacked the capacity (rather than the motivation) to exercise self-control when self-control was described in neurobiological (rather than cognitive) terms.
H3: Participants would attribute less “free choice,” fault and punishment to the impulsive actor when self-control was described in neurobiological (rather than cognitive) terms.

These three hypotheses predict that, contrary to the claims of Greene and Cohen (2004), there is no gap between the intuitive and legal response to neuroscience. The cause of impulsive offending holds retributive significance only when the prior behavior of the offender suggests he lacks a general capacity for self-control; in this context, the lay person may be sensitized to neuroscientific testimony.

## Method

### Design

This study adopted an independent groups design. Two independent variables were manipulated between groups: the normal behavior of the actor (impulsive or controlled) and the source of this behavior (the brain or the self). Finally, a control condition was included that made no reference to self-control. All questions were answered through the online survey platform, Qualtrics.

### Participants

Participants were recruited through Prolific Academic (*N* = 550), earning £0.85 for 10 min of participation, and through Viennese schools (*N* = 271), participating under supervision for free in IT classrooms. Of these 821 participants, 62 (8%) were excluded for incorrectly answering one or more of the questions that checked their basic understanding of the vignette. Of the remaining 759 participants, 55% were female and 43% male (mean age = 30.88, *SD* = 13.33). The most common nationalities were British (47%), Austrian (25%) and American (18%). The most common highest level of completed education was the Bachelor's degree (30%), pre-university college (23%) and high school (18%). Anyone aged 16 or older was eligible to participate. Written informed consent was obtained from all participants.

### Procedure

Participants imagined themselves to be members of a jury in court, yet under the instruction to assume that all the presented information was true. The described offender had already been found guilty; the remaining task was to decide upon an appropriate sentence. First, the participants read an overview of the case, testimony from the prosecutor and testimony from the defense. The offender had attacked his neighbor with a hammer, causing moderate brain injury, after the neighbor's lawnmower launched a stone into the offender's window, cracking it. The offense was described as a “rage attack,” also provoked by the offender discovering flirty text messages from his neighbor on his girlfriend's phone. A full description of the offense can be found in the Appendix, based on Stimulus C used by Schweitzer et al. (2011). To test their comprehension of the case, participants answered basic multiple-choice questions after every page of description and at the end, would be excluded for answering incorrectly.

Participants were randomly allocated to read one of five different defense testimonies. The control testimony stated simply that the offender “admitted his guilt at the earliest opportunity.” The remaining four testimonies additionally described the normal behavior of the offender as either controlled or impulsive, in neurobiological or cognitive terms. The exact text read as follows, with the italicized words manipulated between the four experimental conditions: “*Parts of the brain called the frontal brain lobes/We* normally exercise control over our impulses. An expert psychologist conducted a series of tests on [the offender] to measure how much control *his frontal brain lobes/he* normally *exercise/exercises* over his impulses. // The tests indicated that *[the offender]'s frontal brain lobes/[the offender]* normally *use/uses as much/half as much oxygen/inhibition* as *the average person's frontal brain lobes/the average person*, meaning that [the offender] normally exercises *the same amount/half the amount* of control over his impulses as the average person.” These tests were described as 99% accurate and impossible for the offender to fake.

After reading all the case information, participants decided upon a sentence for the offender to test whether any change in perceptions of the offender impacted the desire to punish the offender; the maximum possible sentence was 16 years, which was described as the actual maximum for this type of offense in England and Wales. This question was then repeated and in this case, participants were also given the opportunity to sentence the offender to a maximum of 16 additional years in secure accommodation; the purpose of the additional time was described as rehabilitation and incapacitation. Participants then indicated their agreement (1 = *strongly disagree*, 6 = *strongly agree*) that: (a) the decision to offend was the offender's fault, (b) the offender would likely reoffend, (c) the offender could be reformed, and (d) the offender could be deterred. Participants who read about the impulsive actor additionally indicated their agreement that: (a) it was the offender's fault that he exerted only half the normal amount of self-control, (b) he was consciously aware that he exerted only half the normal amount of self-control, and (c) it would take him a lot more effort than the average person to exert the normal degree of self-control.

Next, every participant indicated their agreement that: (a) “from the moment the stone cracked the window, [the offender] could have resisted the impulse to attack,” (b) the offender made a conscious decision to offend, (c) “the offender did not really want to attack [the victim]” and (d) “ [the offender]'s decision to attack [the victim] reflected his personality.” Subsequently, participants judged how important (1 = *very unimportant*, 6 = *very important*) each of the following factors were “in causing” the offender to offend: his free choice, evil intent, his brain, his conscious mind, unconscious processes, his emotions, his social life experiences and his genes. Participants who read about the impulsive actor also indicated their agreement that the government should introduce a certain new policy; this policy would require anyone who used less than half the normal amount of self-control to live in secure accommodation until the detained person started to use more than half.

The final set of questions measured belief in biosocial (genetic and environmental) determinism and neurobiological determinism. Specifically, participants indicated their agreement that the offender would have offended, first, if he had the exact same genes and life experiences as the participant, and second, if he had the exact same brain as the participant. Participants also answered these two questions in the reverse direction; reporting their agreement that *they* would have offended, first, if they had the exact same genes and life experiences as the offender, and second, if they had the exact same brain as the offender. Lastly participants indicated the country that represented their nationality, their gender, their age and their highest completed level of education.

## Results

Perceptions of the offender were analyzed using a between-subjects ANOVA, with the Disposition of the offender [Impulsive, Controlled] and its Source [the Self, the Brain] as between-subjects factors; the control condition was not included in this analysis. There were no significant effects on the recommended sentence; Table 1 shows the mean sentences by condition.

**Table 1:** Mean sentence by experimental condition (years).

	**Controlled actor**	**Impulsive actor**
Cognitive testimony	8.31	8.41
Neurobiological testimony	8.62	8.50

### Effects of the disposition

There were main effects of the Disposition on the perceived extent to which the offender: could have resisted the impulse to offend [*F*_(1, 593)_ = 25.09, *p* < 0.001, η_p_^*2*^ = 0.042], could be attributed fault for offending [*F*_(1, 593)_ = 6.63, *p* = 0.010, η_p_^*2*^ = 0.011], would be likely to reoffend without receiving intervention [*F*_(1, 593)_ = 28.29, *p* < 0.001, η_p_^*2*^ = 0.046] and could be reformed [*F*_(1, 593)_ = 11.57, *p* = 0.001, η_p_^*2*^ = 0.019]. Participants were significantly more likely to: attribute fault to the controlled actor (Controlled: *M* = 5.82, *SD* = 1.42; Impulsive: *M* = 5.51, *SD* = 1.46), expect the controlled actor could have resisted the impulse to offend (Controlled: *M* = 5.02, *SD* = 1.07; Impulsive: *M* = 4.58, *SD* = 1.07), and expect the controlled actor could be reformed (Controlled: *M* = 4.48, *SD* = 1.02; Impulsive: *M* = 4.19, *SD* = 1.07). Participants were also less likely to expect the controlled actor to reoffend (Controlled: *M* = 4.00, *SD* = 1.14; Impulsive: *M* = 4.49, *SD* = 1.12).

### Causal attributions

There were main effects of the Disposition on the causal importance attributed to free choice [*F*_(1, 593)_ = 4.85, *p* = 0.028, η_p_^*2*^ = 0.008], social life experiences [*F*_(1, 593)_ = 9.36, *p* = 0.002, η_p_^*2*^ = 0.016], genes [*F*_(1, 593)_ = 29.21, *p* < 0.001, η_p_^*2*^ = 0.047] and the brain [*F*_(1, 593)_ = 16.91, *p* < 0.001, η_p_^*2*^ = 0.028]. In respect to the controlled actor, participants were significantly more likely to attribute his offending to his free choice (Controlled: *M* = 5.65, *SD* = 1.05; Impulsive: *M* = 5.45, *SD* = 1.18) and social life experiences (Controlled: *M* = 5.74, *SD* = 1.10; Impulsive: *M* = 5.45, *SD* = 1.24), yet less likely to attribute his offending to his genes (Controlled: *M* = 3.90, *SD* = 1.29; Impulsive: *M* = 4.50, *SD* = 1.40) and brain (Controlled: *M* = 5.44, *SD* = 1.13; Impulsive: *M* = 5.80, *SD* = 0.98). This effect on the importance attributed to the brain also interacted with the Source of the disposition [*F*_(1, 593)_ = 8.12, *p* = 0.005, η_p_^*2*^ = 0.014]: participants were more likely to attribute the offending of the impulsive actor to the brain only when self-control was attributed to the brain (Controlled: *M* = 5.30, *SD* = 1.15; Impulsive: *M* = 5.90, *SD* = 0.90). Finally, there was a main effect of the Disposition on the belief that the participant would also offend in the same genetic and environmental position as the offender [*F*_(1, 593)_ = 7.18, *p* = 0.008, η_p_^*2*^ = 0.012]: participants were less likely to expect to offend in the genetic and environmental position of the controlled actor (Controlled: *M* = 2.84, *SD* = 1.45) than that of the impulsive actor (Impulsive: *M* = 3.16, *SD* = 1.38).

### Mediation

Mediation analysis was conducted using the PROCESS plug-in for SPSS (Hayes, 2013). There were no indirect effects of the Disposition on fault via the causal importance ascribed to social life experiences (*p* = 0.316), genes (*p* = 0.772) or the brain (*p* = 0.368); since none of these variables predicted fault. There were significant indirect effects of the Disposition on fault via the participant's belief that he/she would offend in the same genetic and environmental position as the offender (β = 0.06, *SE* = 0.03, 95% CI [0.02, 0.13]) and via the causal importance ascribed to free choice (β = 0.07, *SE* = 0.03, 95% CI [0.01, 0.13]). There was no direct effect of the Disposition on fault (*p* = 0.144). Therefore, the greater attribution of fault to the controlled actor was mediated by the participant's belief that he/she would not offend in the same genetic and environmental position and that free choice was more important “in causing” the controlled actor to offend.

### Effects of the source

There were main effects of the Source of self-control on the causal importance attributed to the conscious mind [*F*_(1, 593)_ = 4.37, *p* = 0.037, η_p_^*2*^ = 0.007] and on the belief that the offender did not really want to offend [*F*_(1, 593)_ = 9.93, *p* = 0.002, η_p_^*2*^ = 0.016]. When self-control was attributed to the brain, participants were significantly more likely to attribute the offending to the conscious mind (Brain: *M* = 5.55, *SD* = 0.98; Self: *M* = 5.36, *SD* = 1.20) and less likely to believe the offender did not really want to offend (Brain: *M* = 2.48, *SD* = 1.02; Self: *M* = 2.77, *SD* = 1.21).

### Fault for low self-control

Independent-groups *t*-tests were conducted on the four items that were only asked for the impulsive actor. Participants were less likely to believe that it was the offender's fault that he only used half the normal amount of self-control after reading the neurobiological description (*M* = 2.50, *SD* = 1.16) compared to the cognitive description (*M* = 3.23, *SD* = 1.29), *t*_(299)_ = 5.15, *p* < 0.001, *d* = 0.60.

### The control condition

Independent-groups *t*-tests were conducted to compare the control and experimental conditions. When described in neurobiological terms, people believed the impulsive actor was more likely to reoffend [*t*_(305)_ = 3.73, *p* < 0.001; Exp: *M* = 4.59, *SD* = 1.09; Control: *M* = 4.10, *SD* = 1.21], to have offended because of his genes [*t*_(305)_ = 4.15, *p* < 0.001; Exp: *M* = 4.66, *SD* = 1.41; Control: *M* = 4.01, *SD* = 1.35] and his brain [*t*_(285.75)_ = 4.06, *p* < 0.001; Exp: *M* = 5.90, *SD* = 0.90; Control: *M* = 5.41, *SD* = 1.19], and less likely to have the capacity to resist offending [*t*_(305)_ = 3.24, *p* = 0.001; Exp: *M* = 4.52, *SD* = 1.07; Control: *M* = 4.92, *SD* = 1.11].

## Discussion

People who normally exercise self-control also behave impulsively sometimes—and occasionally that impulsive behavior is criminal. In this study, participants read about a violent offense, committed either by a normally impulsive actor or by a normally controlled actor who experienced a lapse in self-control. The participants were more likely to attribute the criminal actions of the controlled actor to free choice and therefore attribute fault to this offender (H1). Perhaps these participants believed that the controlled actor had freely chosen to deviate from his clearly demonstrated *capacity* for self-control. This finding suggests that lay attributions of blame are informed by the (impulsive or controlled) character of offenders, not only their degree of intentionality (Darley et al., 2000; Carlsmith et al., 2002, 2008; Carlsmith and Sood, 2009; Aharoni and Fridlund, 2012) and their desire to offend (Woolfolk et al., 2006).

Although the controlled actor was attributed more fault, he was not given a longer prison sentence. This null effect on the sentence is most likely explained by the fact that other considerations inform sentencing recommendations; for example, while one would expect the greater attribution of blame to attract a longer sentence, the impulsive offending of the controlled actor was also, by definition, out of character—a factor that typically mitigates punitiveness (Locke and Frankfurt, 1975; Sripada, 2010). Therefore, competing considerations about the character of the offender may have obscured the effect of attributed fault on the recommended sentence.

### How did people explain the offending of the impulsive and controlled actors?

After reading the neuroscientific testimony, participants were more likely to attribute the actions of the impulsive (compared to the controlled or unexplained) actor to his brain and (irrespective of neuroscience) to his genes. This suggests the participants more readily considered biology capable of explaining a stable disposition than a lapse from that disposition (Dar-Nimrod and Heine, 2011); this is perhaps unsurprising, since the vignette only explicitly presented the brain as an explanation of stable disposition. In contrast, the participants were more likely to attribute the actions of the controlled (compared to the impulsive) actor to social life experiences. This suggests the participants more readily considered social influences capable of explaining a deviation from stable character.

From the perspective of neuroscience, social life experiences can only induce impulsive behavior by first influencing brain activity—the “bottleneck” through which every influence on behavior must pass (Greene and Cohen, 2004, p. 1781). In contrast, the current participants perceived the brain and social influences as explanations of character and a lapse in character respectively. Not only is there no scientific basis to this perception of the brain and social influences as in opposition, but the distinction also suggests that people believe in some other mediator of social influence, most likely an independent mind. In this respect, the current study is consistent with prior evidence that people perceive the brain and mind to be separate (Bloom, 2004; Forstmann and Burgmer, 2015). The current study suggests that people also distinguish the factors that inform the basis of brain and mind.

However, the difference in attributions for offending was unable to explain why people attributed more fault to the controlled (than the impulsive) actor. In other words, participants did not derive their attributions of fault from their explanations for the offending. Instead, the difference in fault was explained by two factors: first, the belief that the offending was the inevitable outcome of causal factors (i.e., belief in determinism) and second, the attribution of “free choice” to the offender. In respect to determinism, participants were less likely to perceive the lapse (compared to the disposition) as an *inevitable* product of genetic and environmental influences. Therefore, these participants must have been more likely to attribute the offending of the controlled actor to some third factor that could break the inevitability of causation—presumably a free choice that was not deterministically caused. In turn, this effect partially explained why the controlled actor was blamed more, independent of attributed “free choice.” Hence participants recognized the relevance not of causation but of the *inevitability* of causation, independent of attributed “free choice.” Since the participants therefore considered determinism to be somewhat relevant to blame, their attributions were somewhat incompatibilist.

The increase in fault attributed to the controlled actor was also explained by the greater attribution of “free choice” independent of causal or deterministic beliefs. Hence the retributive significance of “free choice” may not lie in causal or deterministic beliefs, but perhaps instead, in the perceived capacity of the offender to exercise *rational* choice. The participants did not consider the controlled actor more conscious of his choice to offend or the consequences of that choice; therefore, it is unlikely that “free choice” was equated to intentionality (Nahmias et al., 2007; Shepherd, 2012). Instead, the attribution of “free choice” might regard *which part* of the conscious mind the impulse was believed to have bypassed:

“Participants must have thought that even if the agents' conscious mental states weren't bypassed, the agents themselves were; the causally relevant mental states [were]” (De Brigard et al., 2009, p. 10).

The current study proposes that one such “causally relevant mental state” is self-control; more specifically, the capacity to be moved by, and therefore act upon, reason, where reason includes anticipated harm.

The participants may have considered the controlled actor to have a greater capacity to be moved by reason; indeed, he must have been sufficiently moved by reason in the past to exhibit controlled behavior. Therefore, while both offenders wanted to offend, the controlled actor was able to compare his desire to far greater experience of inhibiting desire. Hence the controlled actor could better appreciate the difference between impulsive and considered action and thereby know that this act was excessively impulsive—knowledge that could move the offender to obey the law. Since the offender nevertheless offended, his response appears particularly disproportional to the source of provocation. Legal attributions of responsibility require the offender to know right from wrong (Morse, 2004b), yet the current study provides a new avenue for research: people might also consider the capacity of the offender to know impulsive from considered action and so, disproportional from proportional responses.

### Is a lapse from high self-control freely willed or deterministically caused?

The previously controlled behavior of the controlled actor documents his capacity to exercise rational choice. Since this type of offender clearly meets the legal criterion for responsibility (Morse, 2004b), I expected that participants would no longer be concerned by the *cause* of the lapse in self-control and therefore the neuroscientific testimony (H3). Indeed, there was no effect of neuroscientific testimony. However, this finding cannot be used to suggest people consider neurobiological causation to be irrelevant to responsibility, since the participants were no more likely to make a neurobiological attribution for the lapse after reading neuroscience. In other words, the participants did not consider the lapse in normally controlled behavior to be a lapse *in brain activity*. Presumably, instead, participants believed the controlled actor overrode a default biological tendency to be controlled. It therefore remains a task for future research to assess how people would respond to evidence that the *lapse*, and not only the normal functioning, of self-control was caused by the brain.

### Is the impulsive actor unable or unwilling to exercise self-control?

In contrast to the controlled actor, I expected participants to care far more about *why* the impulsive actor offended. The stability of his impulsive behavior may have primed participants to consider the possibility that the offender lacked the *capacity* to exercise self-control. In turn, neuroscientific testimony may have confirmed those suspicions (H2). Indeed, after reading the neuroscientific testimony, participants were less likely to believe it was the offender's fault that he used only half the normal amount of self-control. However, there was no subsequent reduction in attributions of choice, blame or punishment to the impulsive actor (H3). Therefore, although participants did consider the brain to contain capacities more than motivations, this distinction between capacities and motivations was deemed morally irrelevant.

One could make an alternative interpretation: while evidence of the incapacity (rather than the unwillingness) to exercise self-control might independently reduce attributions of blame for impulsive offending, this effect could be buffered by what neuroscientific evidence suggests about the “real self” of the offender. In a study by Nadelhoffer et al. (in preparation), participants read about an offender who was highly likely to reoffend, as inferred from “brain-based information” or from psychological or statistical information. The participants were more likely to attribute blame, moral responsibility and punishment to the neurobiologically described offender and more likely to attribute his behavior to “his true underlying values and character” (Nadelhoffer et al., 2013, p. 202). Hence neuroscience might actually promote attributions of offending to the “real self” when the science is used to explain a stable disposition to offend; as was the case for the normally impulsive actor in the current study.

The neurobiologically described offender may be perceived similar to the “willing [drug] addict;” as someone who *wants to have* the desire to offend or the desire for the drug respectively (Frankfurt, 1971). In turn, people are more likely to blame offenders who appear to have this meta-desire to offend (Pizarro et al., 2003) and a “real self” that is morally bad (Newman et al., 2014, Study 4). In the current study, therefore, the neurobiologically described offender may have been deemed less responsible for having developed his impulsive disposition, yet more likely to have an impulsive “real self.” Since one would expect the former perception to be mitigating and the latter perception to be aggravating, the two together may have counteracted each other; thereby generating the null effects of neuroscientific testimony for the normally impulsive actor. Though unexpected, the null findings do generalize the results of Appelbaum and Scurich (2014) to neuroscience: their description of impulsive character in *genetic* terms also failed to reduce attributions of moral responsibility to an impulsive offender.

In contrast to prior research, the current vignette explicitly described the stability of the impulsive behavior. Since a stable behavior indicates the possibility of a general incapacity (von Hirsch, 2009), there is a stronger legal argument to examine the source of a stable behavior, as a means of confirming or disconfirming the possibility of incapacity. Although “the distinction between didn't and couldn't is important under the law” (Steinberg, 2009, p. 75) and the current participants made this distinction, it was not ascribed moral significance. This lay perspective represents a highly compatibilist position: the reduced attribution of fault for an impulsive character failed to manifest in a reduced attribution of fault for impulsive offending. This suggests that people may be even more compatibilist than the law, challenging the claim that there exists a “gap between what the law officially cares about and what people really care about” (Greene and Cohen, 2004, p. 1776). The current participants cared even more exclusively about “what the law officially cares about;” that being the capacity to exercise rational choice.

The conclusion is also consistent with the work of experimental philosophy: lay people continue to attribute moral responsibility to actors who develop the desire to deviate from moral decision making for uncontrollable reasons (e.g., Nahmias et al., 2005). People are unconcerned by the ultimate cause of the desire to offend so long as the desire is conscious (Nahmias, 2006; Nahmias et al., 2007; Shepherd, 2012). In the philosophical research, participants are instructed to assume that determinism is true. Yet, even without this instruction, Shariff et al. (2014) found that challenges to free will, including neuroscience that made no reference to determinism, *did* reduce attributions of moral responsibility. Note, however, this finding recently failed to replicate across four studies (Monroe et al., 2017). In fact, even when neuroscience is related to the specific offender, the mock court approach has documented null effects (Schweitzer et al., 2011), conditional effects (Greene and Cahill, 2012) and even reverse effects (Saks et al., 2014). Similarly, the current findings add further doubt to the hypothesized threat of neuroscience (Greene and Cohen, 2004).

This study is unable to specify why attributions of blame and punishment were uninformed by the described cause of the disposition toward impulsivity. In contrast to certain mock court studies (Gurley and Marcus, 2008; Saks et al., 2014), the current study stated the accuracy of the tests of impulsivity (99%). The null effects could therefore reflect the fact that the current participants were implicitly instructed not to infer that the neuroscientific diagnoses were more accurate than the cognitive diagnoses. In other words, neuroscientific testimony might derive influence from its seeming objectivity. However, this interpretation could not explain effects of neuroscientific testimony when the behavioral method of diagnosis remains constant (Schweitzer and Saks, 2011; Schweitzer et al., 2011) or null effects when the method is changed (Schweitzer et al., 2011; Greene and Cahill, 2012). The seductive allure of neuroscience does not appear to be a more general effect of presenting evidence from a “harder” science (Fernandez-Duque et al., 2015). Instead, participants may have feared that if neuroscience could excuse the impulsive actor, every impulsive actor would be excused on the same rationale. Indeed, people are less likely to consider causation relevant to judgements of responsibility when such judgements pose consequences for judgements in real life (Nahmias et al., 2007; Roskies and Nichols, 2008; Shepherd, 2012). In contrast to more severe and so rarer deficits in rationality (e.g., psychosis), mitigation on the basis of impulsive character presents a slippier slope. Hence people may find the far greater applicability of this neuroscience threatening and consequently, in need of resisting.

### How do lay people represent the brain?

Most unexpectedly, participants were *more* likely to attribute impulsive offending to the conscious mind and a “genuine desire” to offend after reading the neuroscientific testimony. This finding could be explained by prior evidence that people believe the brain is responsible for the “core self;” where the “core self” is conceived to include self-control and stable desires (Fernandez-Duque and Schwartz, 2016). The Deep Self model predicts that people attribute greater intent—or in this study, a greater role for the “conscious mind” and “genuine desire”—to behaviors that result from stable dispositions—or in this study, the brain (Locke and Frankfurt, 1975; Sripada, 2010). Similar to the current finding, Saks et al. (2014) found that the presentation of brain scans increased attributions of moral responsibility to schizophrenic offenders.

Although neuroscientific testimony did not increase attributions of fault in the current study, it did increase attributions of the “conscious mind” and “genuine desire.” This finding directly contradicts evidence that people tend to believe the brain bypasses the conscious mind (Nahmias et al., 2007). The discrepancy may reflect the target of explanation: whereas in previous studies, neuroscience was used to explain behavior directly (Nahmias et al., 2007), the current vignette used neuroscience to explain the preceding and clearly conscious process; that is exercising self-control. Therefore, while people may not perceive the brain as conscious by default (Nahmias et al., 2007), this study supports the conclusion of Shepherd (2012): people are willing to adopt this perception, perhaps given their “theory-lite” conception of the relationship between the brain and conscious mind (Nahmias et al., 2014, p. 504).

The current study also challenges the claim that people perceive the brain to be unchangeable and hence, the fear that neuroscience will be used to aggravate sentences (Snead, 2007). We found no impact of neuroscientific testimony on consequentialist concerns, corroborating prior evidence of null effects (Aspinwall et al., 2012; Greene and Cahill, 2012; Saks et al., 2014). The aggravating effect of neuroscience could be more covert; for example, Fuss et al. (2015) found that neurogenetic evidence of psychopathy increased support not for generic preventative detention but for the preventative detention of *irrational* offenders. In contrast, our neuroscientific testimony exerted no effect on support for the preventative detention of (actual or predicted) *impulsive* offenders. There are two potential explanations for the discrepancy between this finding and that of Fuss et al. (2015): neuroscience may only increase the perceived dangerousness of severely irrational offenders (e.g., psychopathic, compared to impulsive, offenders) or *genetic* testimony may be required to induce attributions of dangerousness; indeed, the latter was observed by Appelbaum and Scurich (2014) in judgements of an impulsive murderer. Hence this study questions whether one can generalize to neuroscience the fear that people will make fatalistic inferences about the explicitly caused actor (Miles, 2013), especially the genetically caused actor (Dar-Nimrod and Heine, 2011).

Nevertheless, the described disposition of the offender did influence consequentialist judgements: participants tended to perceive the controlled actor as more capable of resisting offending, less likely to reoffend and more capable of reform. On the former two items, participants also judged the unexplained offender (compared to the neurobiologically impulsive actor) in this same manner. This finding suggests that default perceptions of the unexplained (impulsive) offender resemble those of the controlled actor.

In sum, neuroscience effectively shifted lay conceptions of impulsive character from a failure in the willingness, to a failure in the capacity, to exercise self-control. However, this shift posed no implications for attributions of choice, blame or punishment to the impulsive offender. Yet still, participants attributed greater fault to the normally controlled (than the normally impulsive) offender as a result of compatibilist and incompatibilist concerns; specifically, his perceived degree of dispositional self-control and the inevitability of his criminal behavior.

## Ethics statement

Ethics approval was gained from the Central University Research Ethics Committee at the University of Oxford. All participants provided informed consent.

## Author contributions

TK wrote the method, recruited 280 participants, edited the paper and helped designed the study. RB did the remaining tasks.

### Conflict of interest statement

The authors declare that the research was conducted in the absence of any commercial or financial relationships that could be construed as a potential conflict of interest. The reviewer NE declared a shared affiliation, though no other collaboration, with one of the authors, RB, to the handling Editor.

## Appendix

### The full offense text

Based on Stimulus C (Schweitzer and Saks, 2011)

Overview

Donny Adams, age 32, has been found guilty of Inflicting Grievous Bodily Harm on his neighbor, 38–year–old Robert Samuels.

Mr. Samuels was working in his garden when his lawnmower launched a small stone into Mr. Adams's window, cracking it. The offender, Mr. Adams, immediately ran from his house and struck Mr. Samuels with a hammer, causing him to fall to the ground unconscious.

The emergency hospital doctor found that Mr. Samuels had been knocked out by a single heavy blow to his head, and suffered moderate brain injuries as a result.

[New page] The prosecutor

Mr. Samuels was working in his back garden with a lawnmower, and heard a loud noise as if the lawnmower had hit a stone. After a few seconds, the offender, Mr. Adams, emerged from his house and ran directly at Mr. Samuels while screaming and swinging a hammer.

Mr. Adams already had the hammer in his hand. He had been using the hammer to knock a screw into a wall to hang up a picture at the time the stone cracked his window. Mr. Adams struck Mr. Samuels forcefully in the head with the hammer. Mr. Samuels collapsed to the ground. The offender, Mr. Adams, ranback into his house and closed the door, while two witnesses rang the emergency services.

[New page] The prosecutor (continued) A 'rage attack'

The type of attack committed by Mr. Adams is known as a 'rage attack'. A 'rage attack' is an attack in which the intensity of violence appears to be out of proportion with the intensity of the triggering event (in this case, the window cracking).

The previous day

The prosecutor argued that events the previous day could help explain this 'rage attack'. The day before the stone cracked his window, Mr. Adams checked his girlfriend's phone and found that Mr. Samuels had been sending her flirty messages. By the time the window cracked, Mr. Adams had not confronted his girlfriend or Mr. Samuels about the messages.

Consequently, Mr. Adams became enraged when the stone from Mr. Samuels's lawnmower cracked his window. In retaliation, Mr. Adams attacked Mr. Samuels.

[New page] The prosecutor (continued)

The victim Mr. Samuels sustained moderate brain damage from the forceful blow to his head. He stayed in a coma for 20 days. He has since come out of the coma and returned to his home. However, Mr. Samuels continues to have difficulty remembering many words and controlling fine motor movements (such as holding pencils or typing).

Mr. Samuels is married with 2 young children. He is a teacher in a local high school.
